# Accuracy of doppler assessment of the uterine arteries for the diagnosis of pubertal onset in girls: a scoping review

**DOI:** 10.1038/s41598-023-32880-2

**Published:** 2023-04-08

**Authors:** Amanda Veiga Cheuiche, Candice Moro, Iara Regina Siqueira Lucena, Leila Cristina Pedroso de Paula, Sandra Pinho Silveiro

**Affiliations:** 1 Graduate Program in Medical Science: Endocrinology, Universidade Federal do Rio Grande do Sul, Porto Alegre, RS Brazil; 2 Radiology Division, Hospital de Clínicas de Porto Alegre, Porto Alegre, Brazil; 3 Endocrine Division, Hospital de Clínicas de Porto Alegre, Porto Alegre, Brazil

**Keywords:** Gonadal disorders, Endocrinology

## Abstract

The uterine artery pulsatility index (PI) assessed by Doppler ultrasound reflects the impedance to the blood flow in the vessel distal to the sampling point. We aimed to assess the accuracy of the uterine artery PI for the diagnosis of puberty in girls. A PRISMA-ScR-compliant scoping review was performed in the MEDLINE and Embase databases with the search terms “puberty” and “Doppler ultrasonography”. Studies that included girls aged 0–18 years who underwent pelvic Doppler ultrasound with calculation of uterine artery PI were eligible. Ten studies comprising 1385 girls aged 1.2–18 years were included. The selected studies included participants from Italy, Brazil, Iran, Belgium and Denmark, and were published between 1996 and 2021. Six studies selected girls who were referred for evaluation of pubertal disorders, while four studies included only healthy girls. Nine studies found a significant difference in Doppler signal pattern and PI according to pubertal stage, with PI cutoff points ranging from 2.5 to 4.6 for the diagnosis of puberty, with a sensitivity of 77%–94%, specificity of 85%–100%, and accuracy of 79%–98%. Doppler assessment of the uterine arteries with PI calculation is a useful noninvasive tool in the diagnosis of pubertal onset in girls.

## Introduction

Puberty is a biological maturation process that represents the physical, hormonal, and psychological transition from childhood to adulthood^1^. In girls, the initial manifestation of puberty on physical examination is usually breast development (thelarche), with a normal age range of onset between 8 and 13 years^2^. Therefore, the classic definition of precocious sexual maturation is the development of secondary sexual characteristics before 8 years of age in girls^1^.

The initial laboratory evaluation of precocious sexual maturation in girls includes the measurement of luteinizing hormone (LH), follicle-stimulating hormone (FSH), and estradiol^1^. The difficulties of hormonal assessment include the adequate and standardized determination of normal hormonal concentrations in healthy children, based on age and pubertal stage, for each laboratory method^3^. Additionally, there is a large overlap of estradiol values between pubertal and prepubertal girls^4^. The sensitivity of morning basal LH for the diagnosis of gonadotropin-dependent precocious puberty ranges from 56 to 100%, with a specificity of 64%–100%, depending on the cutoff point and assay used^3^. Therefore, gonadotropin-releasing hormone (GnRH) or long-acting GnRH agonist (GnRHa) stimulation tests are indicated in girls with a non-diagnostic basal LH^5^. The main disadvantages of the GnRHa stimulation test are its high cost, the unavailability of a short-acting GnRH in many countries, the waiting time between blood samples, and the risk of an injection-site reaction.

Pelvic ultrasound is a rapid, noninvasive, and low-cost method to assess uterine development and ovarian volume, with well-established utility in the investigation of pelvic pain, pelvic masses, and atypical genitalia^6^. Additionally, studies have definitively shown increased ovarian and uterine volume in girls with central precocious puberty (CPP) compared with prepubertal controls and girls with isolated premature thelarche (IPT)^7–9^. However, the sensitivity is low for differential diagnosis due to the large overlap of values between groups^10–13^. Doppler ultrasound allows the assessment of utero-ovarian blood flow and flow impedance measurement with the calculation of the pulsatility index (PI), defined as the difference between peak systolic flow and end-diastolic flow divided by the mean flow velocity^14^. The presence of estrogen receptors in the walls of the uterine arteries, which promote a reduction in vascular resistance when there is hormonal stimulation, appears to be responsible for changes in the vascular flow pattern^15,16^. Color Doppler-based studies for the evaluation of female reproductive disorders have highlighted the public health relevance of this safe, efficient, and affordable diagnostic modality^17^.

Within this context, the present study was designed to evaluate the accuracy of the uterine artery PI for the diagnosis of puberty in healthy girls and girls with signs of sexual precocity by means of a scoping review of the literature.

## Methods

### Protocol and registration

This scoping review follows the recommendations of the PRISMA Extension for Scoping Reviews (PRISMA-ScR) protocol^18^. The final protocol was registered prospectively with the Open Science Framework on 15 September 2021^19^.

### Eligibility criteria

We included studies that evaluated the accuracy of Doppler ultrasound with uterine artery PI in girls aged 0 to 18 years for the diagnosis of pubertal onset. The exclusion criteria were studies including pregnant women and review studies. There was no language or date restriction; articles written in languages other than English, Portuguese, and Spanish were considered eligible if they contained sufficient English-language information in the abstract, tables, and figures.

### Information sources

To identify potentially relevant documents, we conducted a systematic search of the MEDLINE (via PubMed) and Embase databases, from inception to January 2022. The final search results were exported into EndNote, and duplicates were removed.

### Search

Comprehensive search queries included descriptors (MeSH and Emtree) based on the expressions “puberty” and “Doppler ultrasound.” We used the following electronic search strategy: ("puberty"[MeSH Terms] OR "puberty"[All Fields] OR "puberties"[All Fields]) AND ("ultrasonography, doppler"[MeSH Terms] OR ("ultrasonography"[All Fields] AND "doppler"[All Fields]) OR "doppler ultrasonography"[All Fields] OR ("doppler"[All Fields] AND "ultrasound"[All Fields]) OR "doppler ultrasound"[All Fields]) for MEDLINE and ('puberty'/exp OR puberty) AND ('doppler ultrasonography'/exp OR 'doppler ultrasonography') for Embase databases.

### Selection of sources of evidence

Two independent reviewers (A.V.C. and C.M.) assessed records for inclusion based on titles and abstracts. Abstracts that did not meet the inclusion criteria or that met the exclusion criteria were discarded. The remaining records and those whose abstracts did not provide sufficient information to decide upon their exclusion were selected for full-text evaluation, which was performed by the same reviewers independently. A third reviewer (S.P.S.) solved any disagreements.

### Data charting process and data items

Two investigators (A.V.C. and C.M.) analyzed the selected studies and extracted data using a standardized system designed for this study. The following information was obtained: first author, year of publication, study design, sample size, inclusion and exclusion criteria, reference method for diagnosis of puberty, age and pubertal stage distribution, PI cutoff point, diagnostic accuracy measures of the index test (sensitivity, specificity, correlation, accuracy, and positive and negative predictive values).

### Synthesis of results

We summarized the information extracted from the included studies in tables.

### Ethics approval

The study protocol was approved by the Hospital de Clínicas de Porto Alegre Research Ethics Committee (GPPG number 2021–0290).

## Results

**Figure 1:**
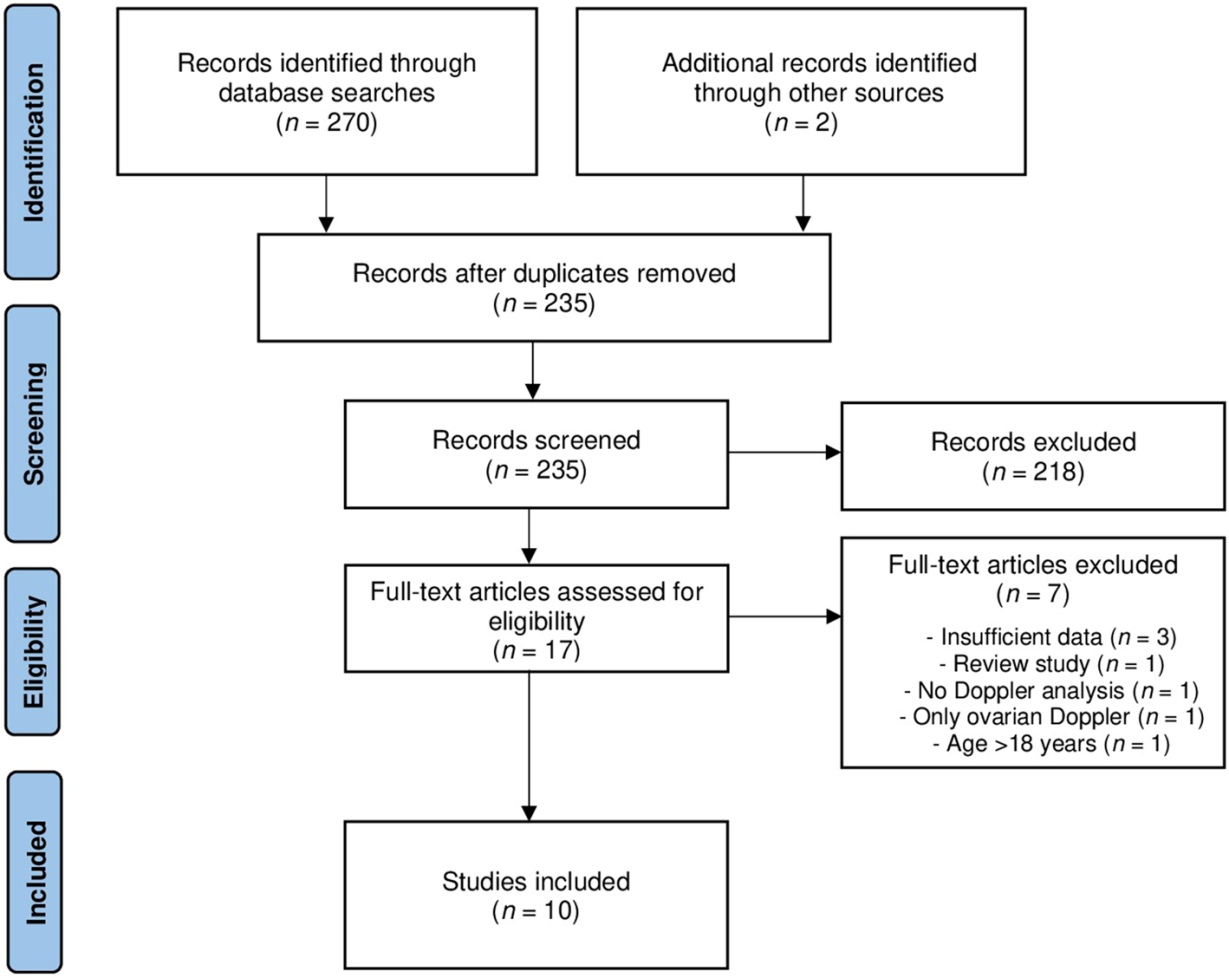
Study flow diagram for the scoping review process.

The search strategy identified 272 citations, of which 235 remained after removal of duplicates. We excluded 218 articles after title and abstract screening, leaving 17 studies for full-text evaluation. Finally, 10 studies including 1385 participants were selected for the scoping review. Figure 1 shows the PRISMA flow diagram.

The selected articles were published between 1996 and 2021 and included girls from five countries (Italy, Brazil, Iran, Belgium, and Denmark). Six studies were cross-sectional in design^20–25^ and four were prospective^26–29^. The ages of the participants ranged from 1.2 to 18 years. The majority of the studies (n = 6) included girls who were referred for evaluation of pubertal disorders, mainly thelarche or pubarche before 8 years of age. Four articles selected healthy girls^20,23,25,29^. Ultrasound examination was reported to be performed by the same radiologist in seven studies^20,21,24–28^, four of which were highly experienced^20,21,24,28^. The main characteristics of the studies are shown in Table 1.

**Table 1:** Characteristics of the included studies.

Author, year, country	Sample Size	Age (Years)	Inclusion criteria	Exclusion criteria	Pubertal stages	Reference test for diagnosis of puberty
Cheuiche, 2021, Brazil	202	5–16 (mean 11.3 ± 1.8)	Girls referred for the evaluation of pubertal development	Thelarche or pubarche before 8 years of age, uncontrolled systemic disease, use of estrogen or progestogen	Prepuberty (n = 39), initial puberty (Tanner breast score 2 and 3; n = 92), final puberty (Tanner breast score 4 and 5; n = 71)	Physical examination (Tanner stage)
Paesano, 2019, Italy	495	1.2–17.2 (mean 8.6 ± 2.2)	Girls with suspicion of pubertal development alterations	GnRH-independent puberty, conditions that can alter puberty, GnRH analog treatment	Prepuberty (n = 207), physiologic puberty (n = 176; 1.7% Tanner breast score 1, 24% score 2, 58% score 3, 15% score 4, 1% score 5), CPP (n = 112; 58% Tanner breast score 2, 38% score 3, 4% score 4)	Presence of at least two of the following parameters: Tanner breast development score $\ge$ 2, LH peak > 5 mU/mL, and longitudinal uterine diameter > 35 mm
Paesano^a^, 2012, Italy	254	11 ± 1 (pubertal), 8 ± 1 (CPP), 8 ± 2 (prepubertal)	Girls referred for evaluation of disorders of pubertal development	Not informed	Disorders of pubertal development (n = 216), CPP (n = 38)	Not informed
Golestani, 2008, Iran	60	8.4 ± 2 (prepuberty), 10.8 ± 1.4 (puberty, without menarche), 13.5 ± 1.9 (puberty, after menarche)	Girls referred for the evaluation of pubertal stages	Chronic disease, large ovarian cysts, polycystic ovaries, GnRH-independent puberty, hormonal therapy	Prepuberty (n = 20), puberty, without menarche (n = 20), puberty, after menarche (n = 20)	Physical examination (Tanner stage)
Battaglia, 2005, Italy	47	7.5 ± 0.3 (girls with normal ovaries), 7.1 ± 0.6 (girls with polycystic-like ovaries)	Girls with GnRH-dependent isosexual precocious puberty and overweight or obesity	Childhood malignancies, chronic diseases, large ovarian cysts, GnRH-independent puberty, isolated premature pubarche, isolated premature thelarche	Tanner breast score 2 or 3 and pubic hair score 2 or 3	Physical examination (Tanner stage) and GnRH stimulation test
Battaglia, 2003, Italy	69	6.4 ± 1.7 (prepubertal), 7.2 ± 0.6 (CPP) 7.0 ± 1.0 (isolated pubarche), 6.3 ± 0.9 (isolated thelarche)	Girls referred for the evaluation of premature breast development and/or pubic hair growth	Chronic disease, large ovarian cysts, polycystic ovaries, GnRH-independent puberty, hormonal therapy	Tanner breast and pubic score 2 with prepubertal response to GnRH test (n = 17), Tanner breast and pubic score 2 with pubertal response to GnRH test (n = 16), isolated pubarche (n = 20), isolated thelarche (n = 16)	Physical examination (Tanner stage), and GnRH stimulation test
Battaglia, 2002, Italy	29	6.6 ± 0.6, 7.3 ± 0.5 (CPP)	Girls referred for the evaluation of premature breast development and/or pubic hair growth	Chronic disease, large ovarian cysts, polycystic ovaries, GnRH-independent puberty, hormonal therapy	Tanner breast score 2 or 3 and pubic hair score 2 or 3; prepubertal response to GnRH test (n = 9), pubertal response to GnRH test (n = 20)	Physical examination (Tanner stage), and GnRH stimulation test
Battaglia, 2002, Italy	27	6.2–7.8	Girls referred for the evaluation of premature pubic hair	Chronic disease, sex steroid-secreting tumors, large ovarian cysts, multifollicular ovaries, gonadal puberty, hormonal therapy	Tanner breast stage 1, Tanner pubic hair stage ≥ 2	Physical examination (Tanner stage), basal hormonal assay
Ziereisen, 2001, Belgium	61	2–15 (mean 10.3)	Healthy female volunteers	Not informed	Tanner breast score 1 (n = 24), Tanner breast score 2 (n = 10), Tanner breast score 3 (n = 11), Tanner breast score 4 (n = 1), Tanner breast score 5 (n = 12), Tanner score not evaluated (3)	Physical examination (Tanner stage)
Laursen, 1996, Denmark	110	6.7–18	Healthy female volunteers	Use of oral contraceptives, menstrual irregularities, gynecological disorders, history of pregnancy	Tanner breast score 1 (n = 31), Tanner breast score 2 (n = 7), Tanner breast score 3 (n = 11), Tanner breast score 4 (n = 25), Tanner breast score 5 (n = 36)	Physical examination (Tanner stage)

^a^Published in conference annals.

**Figure 2:**
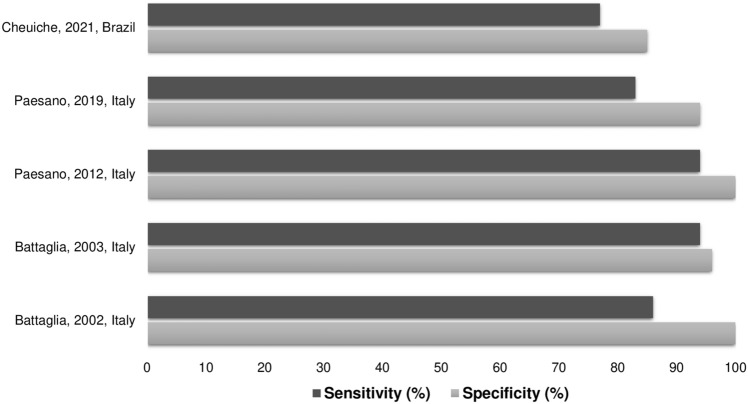
Sensitivity and specificity of the pulsatility index for diagnosis of pubertal onset.

Among the ten included articles, nine found a significant difference in Doppler and PI according to pubertal stage, with PI cutoff points ranging from 2.5 to 4.6 for the diagnosis of puberty onset, with a sensitivity of 77%–94%, specificity of 85%–100% and accuracy of 79%–97%. Figure 2 shows the sensitivity and specificity of the PI for the diagnosis of pubertal onset. Table 2 provides a summary of these results.

**Table 2:** Summary of the main results of the diagnostic value of pulsatility index for the diagnosis of pubertal onset.

Author, year, country	PI Cutoff	Sensitivity (%)	Specificity (%)	PPV (%)	NPV (%)	Accuracy (%)	Other findings
Cheuiche, 2021, Brazil	5.05	77	85	92	61	79	Prepubertal girls had a significantly higher mean PI (6.70 ± 2.15) than girls in initial puberty (4.14 ± 1.55) and those in final puberty (2.81 ± 1.05). Negative correlation of PI with uterine volume, endometrial thickness, uterine longitudinal diameter, and ovarian volumes was detected
Paesano, 2019, Italy	4.60	83	94	95	80	87	The prepubertal group had a significantly higher mean PI (6.3 ± 1.4) than the pubertal group (3.7 ± 1.3)
Paesano^a^, 2012, Italy	4.50	94	100	100	94	97	The prepubertal group had a significantly higher mean PI (6.5 ± 1.57) than the pubertal group (3.2 ± 0.97).
Golestani, 2008, Iran	-	-	-	-	-	-	No significant differences in mean PI were detected between prepubertal girls (1.65 ± 0.29), pubertal girls before menarche (1.64 ± 0.44) and post menarche (1.47 ± 0.25)
Battaglia, 2005, Italy	3.00	-	-	-	-	-	No significant differences in mean PI were detected between girls with normal ovaries (3.0 ± 0.9) and those with polycystic-like ovaries (2.7 ± 0.7). PI was inversely correlated with the LH peak after the GnRH stimulation test
Battaglia, 2003, Italy	2.50	94	96	88	98	96	PI was inversely correlated with FSH, LH and estradiol plasma concentrations. The presence of low PI was more accurate than uterine volume and endometrial echo to detect CPP
Battaglia, 2002, Italy	2.50	86	100	86	100	89	Girls with pubertal response to GnRH had a significantly lower mean PI (2.29 ± 0.19) than those with prepubertal response (3.28 ± 0.37). PI was more accurate than uterine volume and endometrial echo at detecting CPP. PI was inversely correlated with both LH peak and LH/FSH ratio after the GnRH stimulation test
Battaglia, 2002, Italy	–	–	–	–	–	–	Doppler analysis showed elevated resistances within the uterine arteries (mean PI > 3.0) in girls with isolated premature pubarche
Ziereisen, 2001, Belgium	–	–	–	–	–	–	Doppler signal of the uterine arteries displayed three types of flow waves: —Type 1 (narrow systolic flow waves without diastolic flow) was observed in the majority of prepubertal girls, with a mean PI of 6.27 (range 3.5–8.0). —Type 2 (systolic flow waves with interrupted signal during diastole) was detected in five girls with Tanner breast score 1 and five girls with stages 2 or 3, with mean PI of 3.7 (range 2.5–5.0). —Type 3 (broad systolic flow waves with uninterrupted diastolic flow) was observed in post-menarche girls (n = 13) and in girls with Tanner breast stages 2 or 3 (n = 13), with mean PI of 2.0 (range 1.1–2.96). Negative correlation of PI with right ovarian volume, uterine transverse diameter, and uterine length was detected.
Laursen, 1996, Denmark	–	–	–	–	–	–	The PI was similar in Tanner breast stage 1 and 2 (only seven girls in this group), with median of 4.7 (2.7–8.5) and 6.1 (3.0–7.8), respectively. The PI decreased in stage 3 to 2.6 (1.8–8.4) and remained low in stage 4, with median of 2.8 (1.2–7.9), followed by an increase in stage 5 to 3.7 (1.1–6.3). In post-menarche girls, the PI correlated significantly with increasing years after menarche

^a^Published in conference annals.

## Discussion

We performed a scoping review to map the existing literature and to synthesize research evidence about Doppler ultrasound of the uterine arteries for the diagnosis of puberty in girls. Ten articles examined this method in a population of healthy girls at different pubertal stages or those with signs of sexual precocity. Five studies showed that uterine artery PI detected puberty onset with a high accuracy. Additionally, the overwhelming majority of the studies described significant changes in the pattern of uterine artery flow with the progression of puberty. Only one article did not find Doppler ultrasound useful in this setting.

The size and morphology of the uterus and ovaries are relatively stable during childhood, and the uterine fundus and cervix have a similar width^30^. During puberty, the uterus increases progressively in size, becoming wider than the cervix, ovarian volume also increases, and multiple cysts can be seen during each menstrual cycle^31,32^. Ovarian volume and the quantity of large follicles are generally inferior markers of puberty onset compared with uterine parameters^11,33^. On the other hand, a recent systematic review and meta-analysis of 13 studies found that girls with CPP had significantly greater uterine and ovarian measurements, and a uterine length greater than 3.20 cm was a reliable marker to differentiate CPP from IPT with a diagnostic odds ratio of 19.62 and an area under the curve of 0.820^34^. However, the PI was not evaluated in this meta-analysis. Based on the studies identified in our scoping review, the vascular flow pattern in the uterine arteries changes with the growing uterus during pubertal development. There is a decline in vascular resistance with the onset of puberty, expressed by the significantly lower PI at this stage, and finally, a further increase in impedance at the end of puberty, probably by complete uterine angiogenesis.

The cutoff point for uterine artery PI to identify the onset of puberty varies between 2.5 and 5.0 in the literature. This can be explained by differences in the inclusion criteria across studies (only healthy girls vs. girls with physiological puberty or precocious puberty) and by the different reference tests used for defining pubertal onset. Furthermore, the ultrasound examination is operator-dependent, and its outcome can be influenced by the experience of the radiologist. Four studies mention that an experienced radiologist performed the examinations, and in only two articles were the intra- or interobserver variability of the PI measures assessed^20,21^. Finally, the cutoff point is generally given by the optimal cut-point when equal weight is given to sensitivity and specificity^35^. In clinical practice, a higher sensitivity of the PI may be more important for the evaluation of a girl with a suspected pubertal disorder.

Several factors, such as genetics, epigenetics, and lifestyle, are implicated in the onset and completion of puberty^36^. The age at puberty onset varies greatly between different ethnic populations^37^. Studies in the United States using data from the Third National Health and Nutrition Examination Survey showed that non-Hispanic black girls on average enter puberty first, followed by Mexican American and then non-Hispanic white girls, and non-Hispanic black girls had menarche at a substantially younger age^38,39^. Socioeconomic inequalities might also account for important variations in the timing of puberty within and among countries^40^. As an example, in some Asian, African, and South American countries, girls living in privileged conditions also show differences in average menarcheal age as compared with those living in underprivileged conditions^40^. Our scoping review included studies from five countries: three developed economies in Europe, one developing country in Latin America, and one developing country on the Asian continent. Overall, our results showed that Doppler ultrasound performs well in girls of different backgrounds. Due to the well-recognized distinct ethnic and socioeconomic influences on pubertal development, it is important to investigate the accuracy of Doppler ultrasound in each population. In studies that included healthy girls, the mean age of girls with a Tanner breast score of 2 was 9.7 years in the Danish study^25^, that of initial puberty was 10.8 in the Iranian study^23^, that of girls in physiological puberty was 10.5 years in the most recent Italian study^21^, and that of thelarche was 10.2 years in the Brazilian study^20^.

Some limitations of our scoping review must be taken into account. First, the studied population is heterogeneous in relation to both the presence and absence of signs of sexual precocity as well as in the average age of the participants. Nevertheless, scoping reviews are of particular use when a body of literature exhibits a large, complex, or heterogeneous nature not amenable to a more precise systematic review^41^. Second, the sample comprises girls from only five countries, which limits the external validity of the findings. However, the value of scoping reviews in evidence-based practice is the examination of a broader area to identify gaps in the research knowledge base^42^. Third, only five studies reported measures of diagnostic accuracy of the PI. These limitations notwithstanding, we believe we were able to effectively find and summarize the current evidence on the accuracy of uterine artery evaluation in girls.

In conclusion, Doppler assessment of the uterine arteries with PI calculation is a useful noninvasive tool in the differential diagnosis of secondary sexual characteristic onset in girls. Therefore, this parameter should be considered in the diagnostic approach to pubertal disorders, further reinforcing the need for new dynamic collaborations in the development of precision-medicine models. Given the paucity of data on a broader population, future studies following the guidelines for validation of diagnostic methods should be conducted to evaluate the uterine artery PI in girls at different ages and pubertal stages.

## Data Availability

Data and material are available from the authors upon request to the corresponding author.
